# Maternal obesity and intrapartum obstetric complications among pregnant women: Retrospective cohort analysis from medical birth registry in Northern Tanzania

**DOI:** 10.1002/osp4.395

**Published:** 2020-01-13

**Authors:** Amasha H. Mwanamsangu, Michael J. Mahande, Festo S. Mazuguni, Dunstan R. Bishanga, Nickolas Mazuguni, Sia E. Msuya, Dominic Mosha

**Affiliations:** ^1^ Department of Epidemiology and Biostatistics, Institute of Public Health Kilimanjaro Christian Medical University College Moshi Tanzania; ^2^ Department of Community Health Institute of Public Health, Kilimanjaro Christian Medical College Moshi Tanzania; ^3^ Department of Obstetrics and Gynecology Kilimanjaro Christian Medical Centre Moshi Tanzania; ^4^ Department of Monitoring Evaluation and Research Jhpiego Tanzania Dar es Salaam Tanzania; ^5^ School of Public Health and Social Sciences Muhimbili University of Health and Allied Sciences Tanzania

**Keywords:** Body mass index (BMI), intrapartum obstetric complications, maternal obesity, Tanzania

## Abstract

**Background:**

In the last decade, Tanzania has observed a dramatic increase in overweight and obesity among women of childbearing age, a demographic shift that has been associated with intrapartum obstetric complications in high‐income countries. Similar increases in maternal morbidity including postpartum haemorrhage, hypertensive disorders of pregnancy, and rates of caesarean delivery have not yet documented in Tanzania. This analysis describes intrapartum obstetric complications associated with maternal obesity among pregnant women delivering at teaching hospital in Northern Tanzania.

**Methods:**

A retrospective cohort analysis was conducted using the hospital's antenatal care (ANC) and birth registries from 2000 to 2015. The World Health Organization (WHO) body mass index (BMI) categories were applied to classify BMI status of pregnant women within 16 weeks of gestational age at their first ANC visit. Relative risk (RR) of obstetric complications with corresponding 95% confidence intervals (CIs) were estimated using multivariable log‐binomial regression, adjusting for clustering effect for the correlation between multiple deliveries of the same woman.

**Results:**

Among 11 873 women who delivered babies in the hospital during the study period, 3139 (26.5%) fit the definition of overweight and 1464 (12.3%) women with obesity. Compared with women with normal weight, women with obesity were at over 2.6 times at risks of experiencing pre‐eclampsia/eclampsia (RR: 2.66; 95% CI, 2.08‐3.40), pregnancy‐induced hypertension (RR 2.13; 95% CI, 1.26‐3.62), and postpartum haemorrhage (RR 1.22; 95% CI, 1.00‐1.49). Additionally, women with obesity had also higher risk of either elective (RR 2.40; 95% CI, 1.88‐3.06) or emergency (RR: 1.53; 95% CI, 1.34‐1.75) caesarean delivery.

**Conclusion:**

Maternal obesity is an emerging health problem in Tanzania. This study clearly demonstrates an association between increased risk of intrapartum complications and obesity. A review of guidelines around ANC screening and intrapartum care practices considering BMI, as well as appropriate messages for women with obesity, should be considered to improve maternal and newborn outcomes.

## 1 INTRODUCTION

The global prevalence of obesity has increased considerably over the past two decades, and currently, about two billion people are either overweight or obese.^1^ Obesity is a rapidly emerging public health problem for women, with around 17.9% of pregnant women in first trimester presently classed with obesity.^2^ The current increase of prevalence among women with obesity in Africa is steeper than European and Asian countries.^3^ This has a significant public health concern considering the region is also experiencing high prevalence of undernutrition among women of reproductive age.^4^ The new analysis based on data from Demographic and Health Surveys (DHS) from 27 countries in sub‐Saharan Africa (SSA) show that 20% of women with overweight or obesity.^5^ While obesity used to be more prevalent in high‐income countries, since the 2000s, an increase in obesity has been documented in low‐ and middle‐income countries (LMICs), particularly in SSA, where the pooled prevalence of overweight/obesity among general population from 32 SSA countries estimated to be 15.9%.^6^

Tanzania has observed a dramatic increase in trend of women of childbearing age (WCBA) (15‐49 years) who are overweight or obese. The prevalence of overweight or obesity among WCBA in Tanzania increased from 11% in 1992 to 28% in 2016, with higher urban obesity prevalence than rural settings.^7^ Moreover, a household survey conducted in Dar es Salaam revealed an overall prevalence of obesity of 19.2% with women having significantly higher levels of obesity (24.7%) compared with men (9%).^8^

Tanzania is among the countries with the highest maternal mortality ratio (MMR) in SSA. Maternal deaths in Tanzania account for a ratio of 556 per 100 000 women in 2015 to 2016 and represent 18% of all deaths of WCBA.^7^ The main direct causes of maternal death are haemorrhages, infections, unsafe abortions, hypertensive disorders, and obstructed labours.^9^

Previous studies have reported on the association between higher body mass index (BMI) and increased risk of adverse maternal outcomes and obstetric intrapartum complications.^3^, ^10^ These complications include hypertensive disorders in pregnancy such as eclampsia/preeclampsia,^11^, ^12^ gestational diabetes mellitus (GDM),^11^, ^12^, ^13^, ^14^, ^15^, ^16^ thromboembolic disorders,^17^ postpartum haemorrhage (PPH),^19^, ^20^ and maternal deaths^21^, ^22^ as well as services indicative of complications include induction of labour^18^ and caesarean delivery.^19^, ^20^ In addition, the evidence from 14 countries in SSA shows that obesity in pregnancy is associated with increased risks of adverse labour, child, and maternal outcomes.^2^, ^10^

The negative effects of obesity on intrapartum obstetric complications have not yet investigated thoroughly in the LMIC setting. In Tanzania, for example, no studies have assessed the contribution of overweight/obesity status of the mother on obstetric intrapartum complications. Furthermore, Tanzania has adopted focused antenatal care (ANC) guidelines from World Health Organization (WHO), although there is no portion of ANC service devoted for optimal maternity care (clinical guidelines) specific for screening and care for women with obesity. To address this gap, this study describes intrapartum complications associated with early pregnancy maternal obesity among women delivered at a referral hospital in northern Tanzania. Findings could be useful to policy makers, healthcare providers, and program planners who seek to improve obstetric outcomes in Tanzania.

## 2 METHODS

### 2.1 Study design, study area, and study population

The study was a cohort analysis using retrospective record review of women who delivered at Kilimanjaro Christian Medical Centre (KCMC) from January 2000 to December 2015. KCMC hospital is a zonal referral hospital located in Moshi, northern Tanzania, serving a catchment population of over 11 million people, drawn from the Kilimanjaro, Arusha, Manyara, and Tanga regions. An average of 5000 births occurred at KCMC on yearly basis.

**Figure 1:**
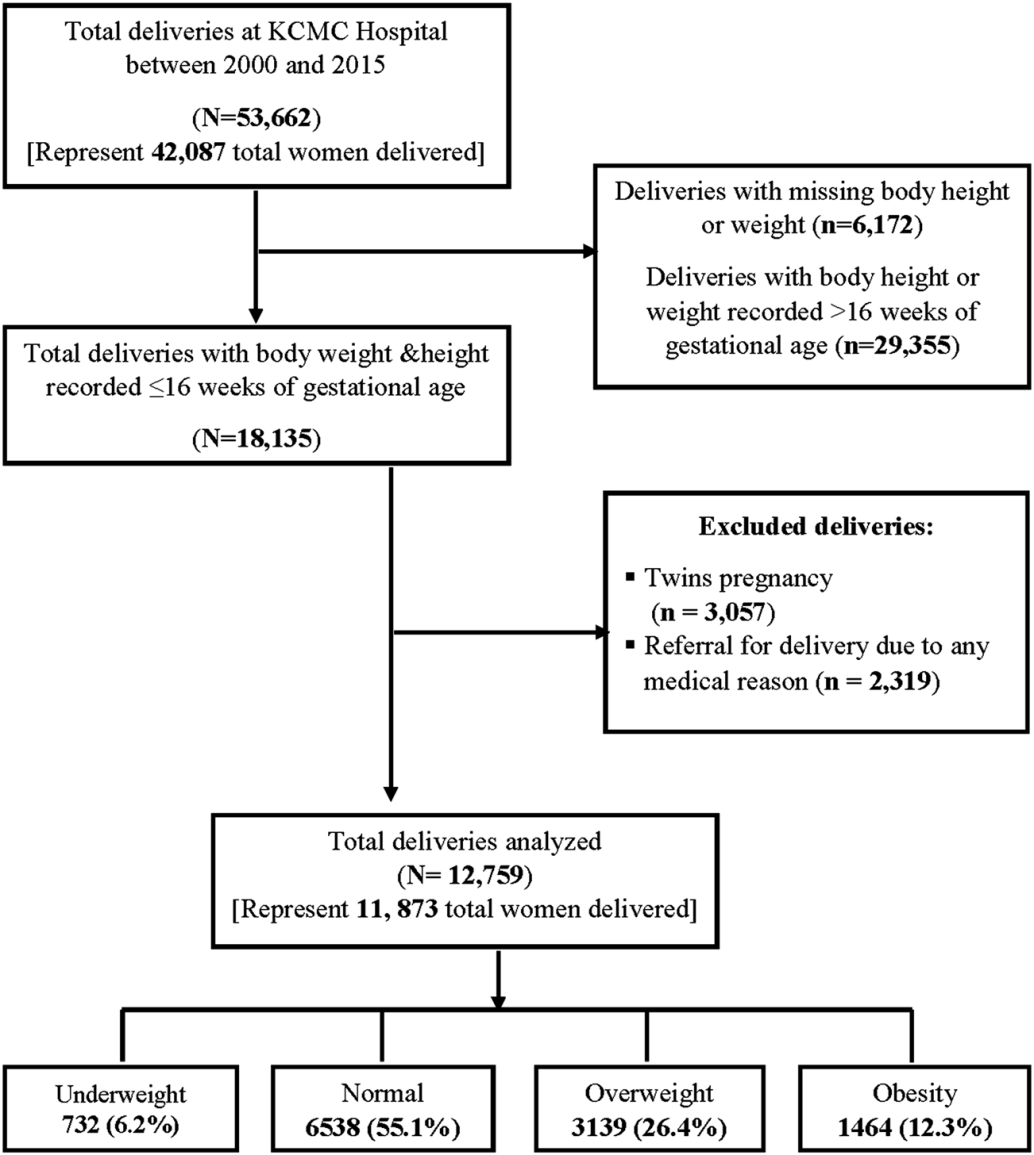
Schematic presentation of the number of deliveries, inclusions, and exclusions during the study period. Data from the Kilimanjaro Christian Medical Centre (KCMC) birth registry

The study population included women who delivered singleton babies at KCMC who had also BMI (both body weight and height) measured at their initial ANC visit. Only women who attended their first ANC visit at most 16 weeks of pregnancy gestational age of were included in the analysis, to exclude women who's BMI might be affected by a later stage of pregnancy. Women with twin pregnancy/multiple gestations (3057) and those referred to the facility for obstetric complications (2319) were excluded from this analysis to minimize overrepresentation of women at high risk of intrapartum complications. Women who delivered more than once at KCMC Hospital during the 15 years period were included in the analysis, and demographic characteristics of these women were described by their first birth. Our final analysed sample was 11 873 of women whom were recorded to have one or more births during the analysis period (Figure 1). These women had 12 759 additional deliveries in the follow‐up period; of them, 11 074 women delivered once, 712 twice, and 87 thrice in KCMC hospital during the analysis period.

### 2.2 Data sources and data set

We extracted data from KCMC birth registry. The birth registry, which has been operational since 2000, is a computerized database, which records information on all deliveries that take place at KCMC hospital. Data from ANC cards and exit interviews are entered into the database within 24 hours of delivery. Information used in this analysis (date of first ANC visit, body weight, and height and date of last menstrual period) are drawn from ANC cards, and information on labour and delivery (mother's weight at admission, date and time of delivery, gestational age, plurality, and mode of delivery) comes from medical registry database. The details of data collection methods and tools have been described elsewhere.^23^, ^24^, ^25^, ^26^ In summary, the data entered in the registry‐included parents' socio‐demographic characteristics, maternal health before and during the current pregnancy, complications during labour and delivery, and regarding the mother's previous pregnancies.

### 2.3 Study variables and variable definitions

The variable on early pregnancy maternal BMI was derived from measured height and weight recorded at the first ANC visit. WHO categories were used to classify BMI: underweight (<18.5), normal weight (18.5‐24.9), overweight (25‐29.9), and obesity (≥30). Levels of education were classified as no education, primary school, secondary school (both ordinary and advanced level), and postsecondary education.

### 2.4 Intrapartum complications and mode of delivery

The mode of delivery was classified as vaginal delivery without complications, caesarean section (elective or emergency), or an assisted delivery (by vacuum or forceps). PPH was defined as blood loss greater than or equal to 500 ml within 24 hours, as visually estimated by the midwife after a vaginal delivery. New onset hypertension of systolic blood pressure (SBP) greater than or equal to 140 mmHg or diastolic blood pressure (DBP) greater than or equal to 90 mm Hg after 20 weeks gestation on two separate occasions was classified as pregnancy‐induced hypertension (PIH). Eclampsia and pre‐eclampsia were analysed in one category of severe hypertensive disorders in pregnancy. Pre‐eclampsia was defined as new onset hypertension accompanied by greater than or equal to 300 mg of proteinuria in urine over 24 hours, measured on two occasions, at least 6 hours apart, after 20 weeks of gestation age in a previously normotensive woman.

### 2.5 Statistical analysis

All analyses were performed using STATA 15 (College Station, Texas, USA). One‐way ANOVA for continuous variables and Pearson's chi‐square test for categorical variables were used to examine socio‐demographic characteristics and intrapartum complications by BMI category. If the number of participants in one or more categories was less than five, Fisher's exact test were applied. The association between maternal BMI and intrapartum complications with risk ratios (RRs) and corresponding 95% confidence interval (CI) were estimated using multivariable log‐binomial regression models, with robust estimation of variances to account for the correlation between repeated observations (multiple deliveries) of the same woman in the analysis period. In multivariable regression analyses, maternal age, number of pregnancies, level of education, marital status, religion, ethical group, and economic activity were confounding factors that were adjusted for. Results were considered to be significant with a *P* value of less than.05. In the log‐binomial regression analyses, underweight and normal weight categories were combined to allow for appropriate numbers of observations per category, with normal weight being a reference category.

### 2.6 Ethical considerations

Ethical approval to carry out the study was provided by the Research and Ethics Committee of Kilimanjaro Christian Medical University College (*Number: 987*). Permission to use data was obtained KCMC medical birth registry administration. The study utilized secondary data that are routinely collected for learning and service delivery; therefore, we did not seek patient consent. To protect women's privacy, confidentiality, and anonymity, we de‐identified client identification biodata during analysis.

## 3 RESULTS

### 3.1 Characteristics and prevalence of maternal obesity among women included in the analysis

Among 11 873 women included in the analysis, mean age of women at their first antenatal visit was 27.3 (SD 5.80) years and mean BMI was 24.4 kg/m^2^ (SD 4.60). The mean gestational age at delivery was 39 weeks (SD 2.39). Majority (62.2%) of the women were residing in urban areas and belonged to Chagga ethnicity (54.2%). Most (89.5%) of the women were married and more than half (51.2%) of the women had primary school education. Furthermore, the majority (67.6%) of the subjects were economically active in the informal sector (Table 1).

**Table 1:** Demographic and socio‐economic characteristics of study sample among women delivered at KCMC Hospital with BMI recorded at ANC before 16 weeks gestational age (N = 11 873)

	Total	Overall	BMI category antenatal booking (Kg/m^2^)				*P* value
			≤18.5 (underweight) N = 732	18.5‐24.9 (normal) N = 6538	25.0‐29.9 (overweight) N = 3139	≥30.0 (obesity) N = 1464	
Maternal characteristics	N	Mean (SD)	Mean (SD)	Mean (SD)	Mean (SD)	Mean (SD)	
Mean age in years (SD)	11 873	27.3 (5.80)	24.4 (5.01)	26.1 (5.52)	28.6 (5.63)	30.8 (5.46)	**<.001** a
BMI at ANC booking (kg/m^2^)	11 873	24.4 (4.60)	17.2 (1.55)	21.9 (1.74)	27.2 (1.39)	33.1 (2.88)	**<.001** a
Gestational age at delivery (weeks)	10 949	39.2 (2.49)	38.9 (2.62)	39.2 (2.49)	39.4 (2.42)	39.2 (2.55)	**.001** a
		**n (%)**	**n (%)**	**n (%)**	**n (%)**	**n (%)**	
**Parity**	11 873						**<.001**
0		6121 (51.6)	497 (67.9)	3755 (57.4)	1380 (44.0)	489 (33.4)	
1‐2		4570 (38.5)	200 (27.3)	2291 (35.1)	1403 (44.7)	676 (46.2)	
≥3		1182 (10.0)	35 (4.8)	492 (7.5)	356 (11.3)	299 (20.4)	
**Education level**	11 863						**<.001**
None		122 (1.0)	11 (1.5)	78 (1.2)	25 (0.8)	8 (0.6)	
Primary		6077 (51.2)	400 (54.7)	3546 (54.3)	1458 (46.5)	673 (46.0)	
Secondary		1482 (12.5)	103 (14.1)	737 (11.3)	431 (13.7)	211 (14.5)	
Tertiary		4182 (35.2)	217 (29.7)	2171 (33.2)	1223 (39.0)	571 (38.9)	
**Current residence**	11 873						**<.001**
Rural		4482 (37.8)	263 (35.9)	2633 (40.3)	1131 (36.0)	455 (31.1)	
Urban		7391 (62.2)	469 (64.1)	3905 (59.7)	2008 (64.0)	1009 (68.9)	
**Marital status**	11 860						**<.001**
Married, cohabiting		10616 (89.5)	624 (85.4)	5793 (88.7)	2849 (90.9)	1350 (92.3)	
Single, widowed, separated		1244 (10.5)	107 (14.6)	738 (11.3)	286 (9.1)	113 (7.7)	
**Religion**	11 802						**<.001**
Christianity		9186 (77.8)	499 (69.1)	5014 (77.2)	2517 (80.5)	1156 (79.2)	
Islam		2616 (22.2)	223 (30.9)	1481 (22.8)	609 (19.5)	303 (20.8)	
**Economic activity**	11 823						**<.001**
Formal employment		2687 (22.7)	121 (16.6)	1341 (20.6)	848 (27.1)	377 (25.8)	
Informally employed		7994 (67.6)	514 (70.6)	4448 (68.3)	2031 (65.0)	1001 (68.6)	
Not economically active		1142 (9.7)	93 (12.8)	724 (11.1)	244 (7.9)	81 (5.6)	
**Gestational age at ANC booking**	11 873						.231
First trimester (<13 weeks)		6691 (56.4)	430 (58.8)	3697 (56.6)	1769 (56.4)	795 (54.3)	
Second trimester (≥13 weeks)		5182 (43.6)	302 (41.2)	2841 (43.4)	1370 (43.6)	669 (45.7)	
**Ethnical groups**	11 873						**<.001**
Chagga		6432 (54.2)	325 (44.4)	3367 (51.5)	1858 (59.2)	882 (60.3)	
Pare		1522 (12.8)	100 (13.7)	853 (13.1)	403 (12.8)	166 (11.3)	
Others		3919 (33.0)	307 (41.9)	2318 (35.4)	878 (28.0)	416 (28.4)	
**Calendar year of delivery**	11 873						**<.001**
2000‐2003		2739 (23.1)	161 (22.0)	1567 (24.0)	726 (23.1)	285 (19.5)	
2004‐2007		2725 (23.0)	167 (22.8)	1543 (23.6)	711 (22.7)	304 (20.8)	
2008‐2011		3591 (30.2)	243 (33.2)	2003 (30.6)	913 (29.1)	432 (29.5)	
2012‐2015		2818 (23.7)	161 (22.0)	1425 (21.8)	789 (25.1)	443 (30.3)	

*Note.* Bold *P* values indicate significant associations.

Abbreviations: ANC, antenatal care; BMI, body mass index; KCMC, Kilimanjaro Christian Medical Centre; SD, standard deviation.

a One‐way ANOVA.

Among 11 873 women included in the study, 732 women (6.2%) were underweight, 6538 (55.1%) had normal weight, 3139 (26.4%) were overweight, and 1464 (12.3%) with obesity. At ANC booking, more than half of women were in the first trimester (up to 13 weeks) of their pregnancy, with 6121 nulliparous (51.6%) and 5752 multiparous (48.4%). Mean age and parity were significantly associated with higher BMI categories (*P* < .001). Married women had higher BMIs compared with their unmarried counterparts (Table 1).

**Figure 2:**
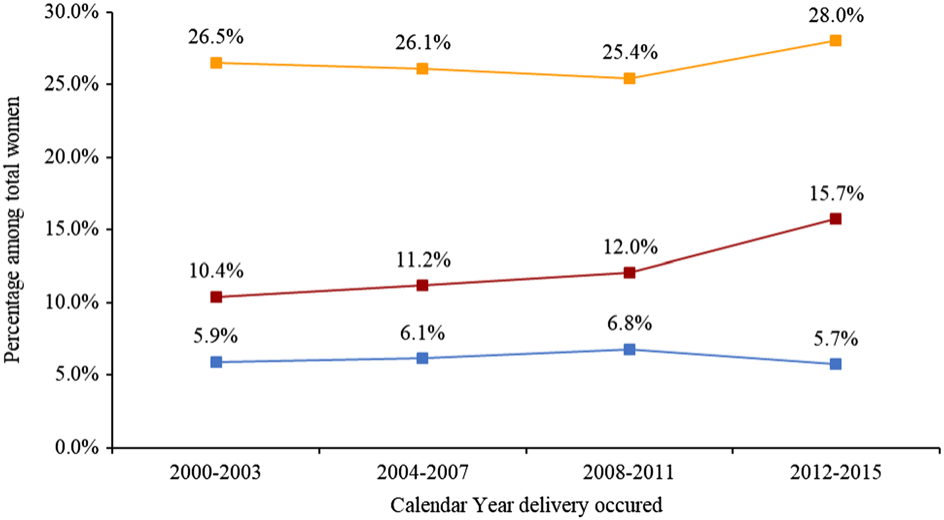
Trends in pregnant women delivered at Kilimanjaro Christian Medical Centre (KCMC) by body mass index (BMI) status. Data from the KCMC birth registry

The prevalence of overweight and underweight women remained virtually unchanged between the 2000 to 2003 and 2012 to 2015. In contrast, the prevalence of obesity increased from 10% in 2000 to 2003 to 16% in 2012 to 2015 (Figure 2). The Cochran‐Armitage test for trend (departures from linearity/goodness‐of‐fit test) shows that the trend is linear (*P* < .001).

### 3.2 Mode of delivery and intrapartum complications by BMI status of the women

About one third 3911 (33.0%) of the women delivered by caesarean section: of these, 86.4% were emergency caesarean sections. The rate of caesarean sections varied significantly between BMI categories (*P* < .001), with higher incidence of caesarean deliveries in women with higher BMI (range 24.7% in underweight women to 43.2% women with obesity). PPH occurred in 942 cases (7.9%), with more PPH occurring among women with higher BMI. PIH occurred more frequently among women with higher BMIs, with the highest incidence of 27 (1.8%) among women with obesity. Four hundred and ninety‐seven cases of preeclampsia/eclampsia were observed with some 128 (8.7%) of the women in the highest BMI category had an increased blood pressure. Sixty‐nine maternal deaths and 46 cases of GDM were observed among studies subgroup during the analysis period (Table 2).

**Table 2:** Maternal death, type of delivery, and intrapartum complication by BMI category among analysed women delivered at KCMC (N = 11 873)

Maternal outcomes	Total	Overall	BMI category at antenatal booking (Kg/m^2^)				*P* value
			≤18.5 N = 732	18.5‐24.9 N = 6538	25.0‐29.9 N = 3139	≥30.0 N = 1464	
	N	n (%)	n (%)	n (%)	n (%)	n (%)	
**Maternal death**	11 873						**<.001**
No		11 804 (99.4)	721 (98.5)	6515 (99.6)	3122 (99.5)	1446 (98.8)	
Yes		69 (0.6)	11 (1.5)	23 (0.4)	17 (0.5)	18 (1.2)	
**Type of delivery**	11 843						**<.001**
Vaginal delivery		7932 (67.0)	548 (75.3)	4548 (69.8)	2006 (64.0)	830 (56.8)	
Caesarean section		3911 (33.0)	180 (24.7)	1972 (30.2)	1127 (36.0)	632 (43.2)	
**Indication of caesarean section**	3911						**<.001**
Elective		533 (13.6)	12 (6.7)	219 (11.1)	170 (15.1)	132 (20.9)	
Emergency		3378 (86.4)	168 (93.3)	1753 (88.9)	957 (84.9)	500 (79.1)	
**Postpartum haemorrhage**	11 873						**.001**
No		10 931 (92.1)	678 (92.6)	6068 (92.8)	2866 (91.3)	1319 (90.1)	
Yes		942 (7.9)	54 (7.4)	470 (7.2)	273 (8.7)	145 (9.9)	
**Preeclampsia/Eclampsia**	11 873						**<.001**
No		11 376 (95.8)	721 (98.5)	6337 (96.9)	2982 (95.0)	1336 (91.3)	
Yes		497 (4.2)	11 (1.5)	201 (3.1)	157 (5.0)	128 (8.7)	
**Pregnancy‐induced hypertension**	11 873						**<.001** ^**a**^
No		11 765 (99.1)	728 (99.5)	6501 (99.4)	3099 (98.7)	1437 (98.2)	
Yes		108 (0.9)	4 (0.5)	37 (0.6)	40 (1.3)	27 (1.8)	
**Gestational diabetes mellitus**	11 873						.292^a^
No		11 827 (99.6)	729 (99.6)	6518 (99.7)	3125 (99.5)	1455 (99.4)	
Yes		46 (0.4)	3 (0.4)	20 (0.3)	14 (0.5)	9 (0.6)	

*Note.* Bold *P* values indicate significant associations.

Abbreviations: BMI, body mass index; KCMC, Kilimanjaro Christian Medical Centre.

Fisher's exact test with n < 5.

### 3.3 Maternal obesity and intrapartum complications

After adjusting for maternal age, parity, mothers education, mothers residence, marital status, religion, economic activity, and ethnical group; compared with normal weight women, overweight women had increased risk of emergency cesarean section (CS) (RR 1.22; 95% CI, 1.11‐1.35), 1.42 fold increased risk of elective caesarean delivery (RR 1.42; 95% CI, 1.14‐1.76), a greater risk of preeclampsia/eclampsia (RR 1.87; 95% CI, 1.87‐2.94), and PIH (RR 1.61; 95% CI, 1.30‐2.01). Women with obesity had more than two‐fold increased risk of delivering by caesarean sections (RR 2.40; 95% CI, 1.88‐3.06), developing PIH (RR 2.13; 95% CI, 1.26‐3.62), and preeclampsia/eclampsia (RR 2.66; 95% CI, 2.08‐3.40) as compared with their counterparts who had normal weight (Table 3).

**Table 3:** Multivariable log‐binomial regression to show the association of BMI category at ANC booking and intrapartum obstetric complications among pregnant women delivered at KCMC hospital, Northern Tanzania (N = 12 759)

Intrapartum obstetric complications	BMI Category at First Antenatal Booking									
	Unadjusted model					Adjusted model				
	<25	25.0‐29.9		≥30		<25	25.0‐29.9		≥30	
	RR [95% CI]	RR [95% CI]	*P* value	RR [95% CI]	*P* value	aRR [95% CI]	aRR [95% CI]	*P* value	aRR [95% CI]	*P* value
Elective CS	Ref	1.87 [1.52‐2.29]	**<.001**	3.51 [2.80‐4.40]	**<.001**	Ref	1.42 [1.14‐1.76]	**.002**	2.40 [1.88‐3.06]	**<.001**
Emergency CS	Ref	1.26 [1.15‐1.39]	**<.001**	1.60 [1.41‐1.81]	**<.001**	Ref	1.22 [1.11‐1.35]	**<.001**	1.53 [1.34‐1.75]	**<.001**
Preeclampsia	Ref	1.74 [1.41‐2.15]	**<.001**	3.19 [2.54‐4.01]	**<.001**	Ref	1.61 [1.30‐2.01]	**<.001**	2.66 [2.08‐3.40]	**<.001**
PPH	Ref	1.22 [1.05‐1.42]	**.010**	1.41 [1.16‐1.71]	**<.001**	Ref	1.13 [0.97‐1.33]	.117	1.22 [1.00‐1.49]	**.050**
PIH	Ref	2.28 [1.47‐3.52]	**<.001**	3.32 [2.03‐5.41]	**<.001**	Ref	1.87 [1.19‐2.94]	**.007**	2.13 [1.26‐3.62]	**.005**

Adjusted model: adjusted for maternal age, parity, mothers education, mothers residence, marital status, religion, economic activity, and ethnical group. Bold *P* values indicates significant associations.

Abbreviations: ANC, antenatal care; aRR, adjusted risk ratio; BMI, body mass index; CI, confidence interval; KCMC, Kilimanjaro Christian Medical Centre; PIH, pregnancy‐induced hypertension; PPH, postpartum haemorrhage; RR, relative risk.

## 4 DISCUSSION

The findings of this study indicate that about a quarter and one tenth of pregnant women who delivered at KCMC were overweight and obese, respectively. The intrapartum complications associated with maternal obesity were preeclampsia, PIH, and PPH as well as elective and emergency caesarean section delivery. These prevalence findings are similar to those presented by Onubi et al in a meta‐analysis of 13 SSA countries,^2^ and slightly lower than seen in studies in Ghana^10^ Sudan^27^ and South Africa.^28^ Discrepancies in prevalence of maternal obesity between the current analysis and other published findings from African countries could be explained by differences in the study design and sample size. The study conducted in Ghana had a shorter spanning time frame and had a smaller sample size, possibly leading to an overestimation of prevalence. The prevalence of obesity observed in our study was also lower compared with the studies in Europe,^19^, ^29^ Asia,^30^ and Australia.^31^ This may concur with the reported upsurge of maternal obesity prevalence in SSA.^2^

Differences in prevalence between high‐income countries and low‐income countries is more likely to be attributed by population health differences and awareness of risk factors of obesity. High‐income countries have facilities and programs to address obesity in both pregnant and nonpregnant women, and some are incorporated into ANC services.^32^ The differences might also be attributed the timing of attendance at ANC during the first trimester of pregnancy. Data are scarce on proportions of women attending their first ANC visit within 4 months of pregnancy in Kilimanjaro region, but generally, in Tanzania, about a quarter of women attend their first ANC visit, which is lower compared with other countries.^7^, ^10^ KCMC primarily serves the population of Kilimanjaro region, where about 98% of pregnant women attend ANC and 91% deliver in the health facilities.^7^ Because the current analysis only included women who were less than or equal to 16 weeks of gestational age at first ANC visit, only a quarter (24%; 12 759/53 662) of the total deliveries that occurred at KCMC in the study period were eligible for inclusion. To rule out the differences in our group compared with the whole, we conducted subgroup analysis among the women included and excluded from the study and found no difference in terms of maternal age, education level, parity, area of residence, and marital status.

In our study, women with obesity were at increased risk of eclampsia/preeclampsia. This is similar to previous studies, which reported an association between pre‐pregnancy obesity and preeclampsia, reportedly due to high fat composition among women with obesity.^2^, ^18^, ^19^, ^29^, ^31^, ^33^, ^34^, ^35^ These findings underscore the importance of frequent monitoring blood pressure during prenatal/ANC since the first sign of preeclampsia is commonly a rise in blood pressure, calling for strengthening routine blood pressure checks in ANC services keeping BMI categories in mind.

This study revealed that maternal overweight and obesity is a potential screening risk factor for PPH during delivery. This is in agreement with the previous studies, which elicited PPH as a pregnancy complication associated with maternal obesity.^36^, ^37^ Whereas a cohort study conducted in Denmark found that PPH was unaffected by BMI,^40^ studies from the Netherlands, Brazil, and Oman revealed that women with obesity were more likely to have PPH as compared with women with normal weight.^30^, ^38^, ^39^

In this study, we found that women with obesity had increased risk of caesarean deliveries compared with normal‐weight women. Similarly, studies elsewhere have shown that overweight or women with obesity had an elevated risk of both elective and emergency caesarean deliveries.^10^, ^18^, ^19^, ^40^, ^41^ This could be explained by comorbid conditions such as diabetes mellitus, high blood pressure, preeclampsia, foetal presentation, and macrosomia of the baby. The higher frequency of emergency caesarean deliveries is a major concern due to complications associated with recurrence of caesarean deliveries in next pregnancies. Although this study conducted at zonal referral hospital, which would be expected to have more complicated cases, which need emergence caesarean deliveries, but during analysis, we tried to minimize selection bias by excluding all referrals. Therefore, guidelines around intrapartum care in Tanzania might be more appropriate to today's obstetric population if they were tailored to address obesity in pregnancy and particularly caesarean deliveries among women with obesity.

Although we did not have data available to identify the primary cause of PPH, pre‐eclampsia, and indication of CS, the study findings suggest obesity as one risk factor for these conditions. PPH and pre‐eclampsia are among the major causes of maternal death in majority of developing countries including Tanzania.^6^ Therefore, these findings underscore the need to incorporate clinical guidance around obesity and overweight into clinical guidance for healthcare providers providing both ANC and intrapartum care in Tanzania. The findings presented here could be useful for policy makers and practitioners seeking to improve the quality of ANC and intrapartum care in Tanzania or similar LMIC settings that are experiencing increase in prevalence of women with overweight or obesity.

## 5 CONCLUSION

Maternal obesity is an emerging health problem in Tanzania with increased risks of adverse pregnancy outcomes. This has implications around awareness of maternal obesity by both women with pregnancy intentions or who are pregnant, as well as healthcare providers. ANC services should utilize client information on BMI as part of management of the pregnant women. Additionally, policy makers may consider revisiting or revising ANC and intrapartum care guidelines to reflect additional risk among women with obesity.

## AUTHOR CONTRIBUTIONS

Conceptualization: Amasha Mwanamsangu and Dominic Mosha

Data curation: Amasha Mwanamsangu and Festo S. Mazuguni

Formal analysis: Amasha Mwanamsangu and Festo S. Mazuguni

Investigation: Michael J. Mahande, Sia E. Msuya, and Dominic Mosha

Methodology: Dunstan Bishanga and Nickolas Mazuguni

Project administration: Dominic Mosha and Michael J. Mahande

Supervision: Dominic Mosha, Michael J. Mahande, and Sia E. Msuya

Writing ± original draft: Amasha H. Mwanamsangu, Dominic Mosha, and Festo S. Mazuguni

Writing ± review and editing: Sia E. Msuya, Dunstan R. Bishanga, and Nickolas Mazuguni

## Data Availability

The Kilimanjaro Christian Medical Centre owns the medical birth registry data. Data requests can be sent to Dr Gillard Massenga (Executive Director of the Kilimanjaro Christian Medical Centre) at drgmasenga@gmail.com subject to obtaining clearance from the Medical Research Coordinating Committee (MRCC) of the National Institute of Medical Research of Tanzania. For information on how to obtain clearance to access the data, contact the MRCC at 255‐22‐2121400 or headquarters@nimr.or.tz.
